# The Role of Nutrition in the Prevention and Management of Bronchopulmonary Dysplasia: A Literature Review and Clinical Approach

**DOI:** 10.3390/ijerph18126245

**Published:** 2021-06-09

**Authors:** Gustavo Rocha, Hercília Guimarães, Luís Pereira-da-Silva

**Affiliations:** 1 Department of Neonatology, Centro Hospitalar Universitário de São João, 4200-319 Porto, Portugal; herciliaguimaraes@gmail.com; 2 Department of Pediatrics, Faculdade de Medicina da Universidade do Porto, 4200-319 Porto, Portugal; 3 Comprehensive Health Research Centre (CHRC), NOVA Medical School|Faculdade de Ciências Médicas, Universidade Nova de Lisboa, 1169-056 Lisbon, Portugal; l.pereira.silva@nms.unl.pt; 4 Neonatal Intensive Care Unit, Hospital Dona Estefânia, Centro Hospitalar Universitário de Lisboa Central, 1169-045 Lisbon, Portugal

**Keywords:** bronchopulmonary dysplasia, enriched formulas, fluid restriction, growth monitoring, human milk fortification, preterm infants

## Abstract

Bronchopulmonary dysplasia (BPD) remains the most common severe complication of preterm birth, and nutrition plays a crucial role in lung growth and repair. A practical nutritional approach for infants at risk of BPD or with established BPD is provided based on a comprehensive literature review. Ideally, infants with BPD should receive a fluid intake of not more than 135–150 mL/kg/day and an energy intake of 120–150 kcal/kg/day. Providing high energy in low volume remains a challenge and is the main cause of growth restriction in these infants. They need a nutritional strategy that encompasses early aggressive parenteral nutrition and the initiation of concentrated feedings of energy and nutrients. The order of priority is fortified mother’s own milk, followed by fortified donor milk and preterm enriched formulas. Functional nutrient supplements with a potential protective role against BPD are revisited, despite the limited evidence of their efficacy. Specialized nutritional strategies may be necessary to overcome difficulties common in BPD infants, such as gastroesophageal reflux and poorly coordinated feeding. Planning nutrition support after discharge requires a multidisciplinary approach to deal with multiple potential problems. Regular monitoring based on anthropometry and biochemical markers is needed to guide the nutritional intervention.

## 1. Introduction

Bronchopulmonary dysplasia (BPD), also named chronic lung disease of prematurity, is a lung disease that causes dependence on oxygen for an extended period of time [1,2,3]. The “new BPD” seen today results from a reparative process in alveolar and vascular compartments of the lung, after injury caused by ante- and postnatal pathogenic factors leading to reduced, large, thin-walled alveoli, and less fibrosis when compared to the “old BPD” [2].

The definition of BPD has been a challenging issue. In 2001, a workshop sponsored by the National Heart, Lung, and Blood Institute defined BPD as the persistence of oxygen requirement at 28 days of life and 36 weeks postmenstrual age (PMA) [4]. A more recent Eunice Kennedy National Institute of Child Health and Human Development workshop proposed some refinements to the 2001 definition of BPD [5]. 

The overall incidence of BPD in infants delivered below 28 weeks’ gestational age is about 30–68% and is inversely proportional to the gestational age [6]. The large variability in rates among centers is partially related to differences in clinical practices, such as the criteria used for the management of mechanical ventilation [7]. 

BPD remains the most common severe complication of preterm birth and is characterized by a high grade of inflammation of the immature lung [8,9]. It represents a major challenge for neonatologists who deal with modifiable risk factors for BPD development, including surfactant replacement therapy, ventilation strategies, corticosteroids, inhaled nitric oxide, inhaled bronchodilators, and macrolides [10,11]. Among postnatal factors, nutrition plays a central role in lung growth and repair [1,12,13]. Nevertheless, it should be kept in mind that malnutrition may start in intrauterine life, constituting an important prenatal risk factor for BPD [14,15,16,17,18]. After preterm birth, several problems associated with extreme immaturity result in difficulty in achieving sufficient energy and nutrient intake [19]. A recent study by Milanesi et al. [20] found that preterm infants who developed BPD, compared with those without BPD, received an energy/protein ratio below what is recommended. A retrospective cohort study reported that, in extremely preterm infants, high fluid intake containing low energy during the first postnatal week is associated with the severity of BPD [20]. Along the same lines, other studies have described the association of postnatal deficit in energy and nutrient and postnatal growth restriction with the development of BPD [21,22,23,24]. 

## 2. Aim

This narrative review aimed to gather evidence on the nutritional support of preterm infants with increased risk for BPD, or with established BPD, in order to provide data to optimize their nutritional approach.

## 3. Literature Search

An extensive search in the databases of medical literature, including Pubmed/Medline, Scielo, Medscape, Cochrane Database of Systematic Reviews, Cinahl Complete, and ClinicalTrials.gov, was performed, including the years from 1980 to 2021 as well as information available from Google. The terms “antioxidants”, “bronchopulmonary dysplasia”, “chronic lung disease”, “enteral nutrition”, “functional nutrients”, “morbidity”, “nutritional management”, “parenteral nutrition”, “prematurity”, and “preterm infant” were used for the search.

## 4. Fluid Management in Infants at Risk for and with Established BPD

Preterm neonates with risk factors for the development of BPD should be considered high-risk infants. Compared to full-term neonates, very low birth weight (VLBW) and extremely low birth weight (ELBW) infants have a higher proportion of body water, more immature renal function, and a limited capacity to eliminate excess of fluids [25]. A physiological contraction of extracellular fluid, with a negative water and sodium balance, occurs during the first postnatal week [25]. Excessive fluid intake will impair this physiological mechanism and is associated with a significant risk of hemodynamically significant patent ductus arteriosus [26]. Moreover, an early fluid overload (150–190 mL/kg/day) [27] may cause pulmonary edema, with reduction of lung compliance, increased airway resistance, and the need for more aggressive respiratory support [28,29,30]. 

In a retrospective cohort study of ELBW infants, it was found that high fluid intake during the first postnatal week and lack of physiological weight loss in the first 10 postnatal days were associated with an increased risk of BPD [31]. A meta-analysis of randomized controlled studies determining the effect of fluid intake on morbidities and mortality in premature infants concluded that the risk of BPD was not significantly affected by water intake, although fluid restriction was associated with a trend toward reduced risk of BPD [31]. Thus, it seems prudent to employ a strategy of fluid restriction in preterm infants considered to be at high risk for BPD. A reasonable approach is to start with fluid intake not exceeding 80–100 mL/kg in the first postnatal day, with a subsequent progressive increase, up to a maximum of 135–150 mL/kg/day [31,32,33]. In infants with established BPD, a restriction of 135 mL/kg/day, or less, may be necessary in severe cases [31,32,34]. In this regard, fluid restriction should be within the clinically acceptable limits of fluid intake, since reduction of free water intake may lead to an increase in renal solute load with the risks of renal dysfunction and nephrocalcinosis [33]. When fluid restriction is prescribed, evaluation of net fluid balance is mandatory, which includes the monitoring of body weight, urine output, plasma sodium, blood pressure, and functional echocardiographic assessment [33,34].

Body temperature and environmental humidity influence the fluids policy [35,36,37,38,39]. The temperature of the abdominal skin should be kept between 36.0 °C and 36.5 °C, and the inspired air temperature (hood, CPAP, or ventilator) between 34.0 °C and 41.0 °C at the Y-piece, with a relative humidity of 100% [35,36,37,38,39]. A survey on incubator humidity practices in the management of preterm infants found a wide variation in humidification practices, but the majority of the surveyed centers used a starting humidity higher than 80% [38]. A systematic review including 12 studies assessing preterm infants’ outcomes related to incubator humidity concluded that 60–70% humidity in the first postnatal week was enough to reduce the transepidermal water loss in infants born at 26 weeks or older; however, more research is needed regarding more immature infants [37]. 

Phototherapy may increase insensible water loss and addition of 10–20 mL/kg/day to the total fluids may be needed, although this seems unnecessary when using the newer phototherapy lamps with light-emitting diodes [40].

## 5. Nutritional Requirements in Infants with BPD

Nutrition plays a critical role in the prevention and management in infants with BPD, and a vicious cycle may occur. Growth failure in BPD infants is predominantly due to malnutrition. Malnutrition, in turn, seems to worsen BPD probably by compromising lung development and function, and feeding difficulties in these infants can further affect nutrition [12,41].

As BPD is not typically diagnosed until 28 days of life or 36 weeks PMA, attempts to prevent postnatal growth failure cannot be made soon enough, as this condition can result in nutrient deficits that may be difficult to recover from [12]. 

In BPD infants, a status of increased respiratory work and inflammatory response, along with the lung damage/repair process, is characterized by higher energy consumption [35]. This energy should be provided in restricted fluid intake, since fluid overload may cause pulmonary edema, which can decrease lung compliance and increase airway resistance [14].

Uberos et al. [41], in a cohort study, found that infants who developed BPD, compared with those who did not, received a lower total intake of energy (76.1 vs. 91.1 kcal/kg/day), carbohydrate (11.6 vs. 12.6 g/kg/day), and fat (2.5 vs. 3.4 g/kg/day) during the first 14 days of life. Klevebro et al. [42] examined the effect of early nutritional intake on growth and the risk of BPD in 296 extremely preterm infants and found that, between days 7 and 27, every additional 10 kcal/kg/day in energy intake was associated with a 9% reduction in the risk of BPD. 

Improved nutritional strategies have enhanced postnatal growth in infants at high risk of growth restriction [43]. Nevertheless, the optimal energy intake for children at risk of BPD or with established BPD is still to be defined. It seems that infants with confirmed BPD should receive an energy intake in a range of 120–150 kcal/kg/day and a protein intake of at least 3.5 g/kg/day [44]. In infants with chronic lung disease, a high-fat diet theoretically produces lower rates of carbon dioxide production than a diet with a lower fat and higher carbohydrate content [45]. However, pulmonary function test results were found to be equivalent in infants receiving high-fat or high-carbohydrate feedings [46]. In addition, 65% of nonprotein energy supplied as carbohydrate was found to be more effective than energy supplied as fat in sparing protein oxidation in enterally fed LBW infants [47].

In healthy preterm infants receiving adequate nutrition support, an optimal weight gain velocity of 15–20 g/kg/day is expected [48].

## 6. Functional Nutrients with Potential Beneficial Effects on BPD

The preterm infant’s inability to down-regulate and maintain control of the inflammatory immune response may facilitate ongoing lung damage, leading to a chronic inflammatory state [49]. Some functional nutrients with antioxidant effects could play a role in reducing lung inflammation [49,50,51]. Despite the lack or limited evidence of their protective effect against BPD through an antioxidant effect or other mechanisms, their use with this purpose, as supplements in parenteral and enteral nutrition, is revisited.

### 6.1. Polyunsaturated Fatty Acids

In a historical cohort study of preterm infants with less than 30 weeks of gestation, a rapid decline in DHA and arachidonic acid levels in the first postnatal week, with a concomitant increase in linoleic acid (LA) levels, was observed [52]. Increased risk of BPD was associated with decreased DHA levels (OR 2.5; 95% CI 1.3–5.0) and increased LA:DHA ratio (OR 8.6; 95% CI 1.4–53.1) [52]. Based on data from preterm infants and animal studies suggesting that docosahexaenoic acid (DHA) serves as a general preventive agent against inflammation, observational and interventional clinical studies were developed to improve DHA delivery in preterm infants to reduce the risk of BPD [49]. In a multicenter, randomized controlled trial, breast milk-fed, very preterm infants with birth weight <1250 g born to mothers who were given a high-DHA diet had a 12% decrease in the incidence of BPD compared with infants whose mothers did not receive supplements [50]. This effect was not observed in other clinical trials of breast milk-fed infants born at less than 29 weeks’ gestation to mothers who were given DHA during the neonatal period, compared with the placebo. However, these results may have been affected by the early trial termination [53]. In another trial, it was reported that infants born at less than 29 weeks’ gestation and receiving enteral emulsion providing 60 mg/kg/day of DHA, from the beginning of enteral feeding until 36 weeks PMA, did not have a lower risk of BPD than those given a control emulsion [54]. A meta-analysis including 14 randomized controlled trials that involved 3531 preterm infants investigated the efficacy of intervention with n-3 polyunsaturated fatty acids on the incidence of BPD and found no evidence (risk ratio 0.99; 95% CI 0.84–1.18) to support this intervention [55]. A similar conclusion was obtained in another systematic review and meta-analysis of four randomized controlled trials involving 1966 very preterm infants [56].

### 6.2. Amino Acids

Glutamine during oxidative stress can reduce cell injury by increasing glutathione [57]. This finding motivated supplementation of this amino acid in preterm infants. However, a meta-analysis of 11 randomized controlled trials including 2771 preterm infants concluded that parenteral and/or enteral supplementation with glutamine did not decrease BPD [58]. 

N-acetylcysteine is a precursor of cysteine and is itself a free radical scavenger [33]. A multicenter, double-blind trial showed that a six-day course of intravenous N-acetylcysteine infusion during the first postnatal week did not prevent BPD in ELBW infants [59].

### 6.3. Vitamins

Vitamin A regulates the growth and differentiation of epithelial cells in the respiratory tract and is an important radical-scavenging antioxidant [60,61]. ELBW infants have low plasma and tissue concentrations of vitamin A, and vitamin A deficiency may predispose to BPD [62]. Clinical trials showed that intramuscular administration of vitamin A decreased the risk of BPD in ELBW infants [62,63]. Based on these results, it was questioned whether a higher dose of vitamin A supplementation might further reduce the risk of BPD [64,65]. Meanwhile, a systematic review and meta-analysis of four studies including 1011 preterm infants concluded that vitamin A supplementation for ELBW infants benefited oxygen dependency at 36 weeks PMA in survivors (pooled risk ratio, 0.88; 95% CI 0.77–0.99) [66]. Another systematic review and meta-analysis of three studies including 612 preterm infants assessing the effect of enteral vitamin A supplementation on BPD found a significant reduction in the incidence of BPD in the vitamin A-treated group compared to placebo (OR 0.57; 95% CI 0.24–1.35) [61]. 

Vitamin E is another radical-scavenging antioxidant that protects cell membranes from oxidative injury [67]. Tocopherol deficiency worsens the effect of oxygen toxicity on lung tissue and its supplementation may have a protective effect on premature lungs [33]. A strong correlation of BPD with low plasma vitamin E and selenium levels measured in cord plasma and on the third postnatal day was reported in premature infants [51]. However, in preterm infants it was not demonstrated that supplemental vitamin E during the acute phase of therapy for respiratory distress syndrome modified the development of BPD [68].

Inositol, as a member of the vitamin B complex, is involved in surfactant synthesis and maturation, and its serum levels are low in preterm infants [33]. A meta-analysis evaluating the effect of inositol supplementation in preterm infants found a trend toward the reduction of BPD at 28 days, but this did not reach statistical significance [69].

### 6.4. Trace Elements

Copper, zinc, selenium, and manganese act as co-factors of antioxidant enzymes, such as the copper–zinc superoxide dismutase and manganese superoxide dismutase [33,70]. However, in ELBW infants, an association between BPD and decreased antioxidant enzyme activities related to trace elements was not demonstrated [71]. 

In a prospective study including 83 preterm infants, it was found that BPD was independently associated with significantly lower serum zinc levels at term age than those who did not develop the disease [72]. A randomized controlled trial including 193 VLBW infants determined the efficacy of zinc supplementation in reducing morbidity. No difference was observed in the rate of BPD, necrotizing enterocolitis, sepsis, periventricular leukomalacia, or retinopathy of prematurity between the group receiving zinc supplementation and that not supplemented. However, the risk of developing at least one of these morbidities was significantly reduced (OR 0.513; 95% CI 0.280–0.939) in the supplemented group [73].

The reduced stores of selenium in preterm infants motivated supplementation, but a meta-analysis of three trials found no benefit of selenium supplementation in the development of BPD [74].

## 7. Nutritional Approach in the Prevention and Management of BPD

Table 1 summarizes the preventive nutritional approach in infants at high risk of BPD and Table 2 summarizes nutritional management in infants with established BPD, using parenteral and/or enteral nutrition either in the hospital or after discharge.

**Figure 1:**
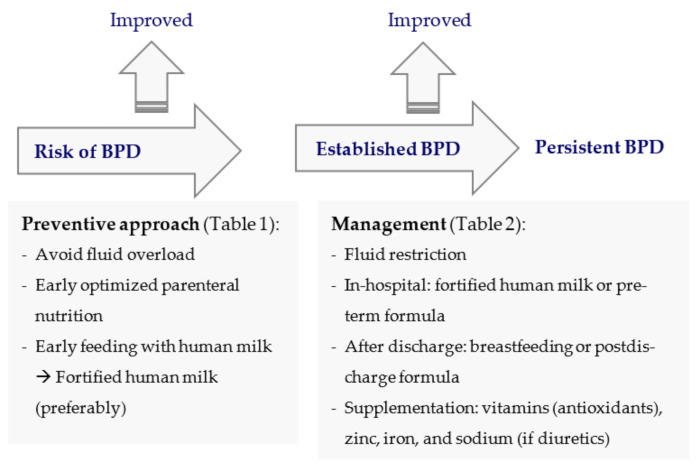
Preventive nutritional approach in infants at high risk of BPD and nutritional management in infants with established BPD. BPD: bronchopulmonary dysplasia.

Figure 1 schematically represents the nutritional approaches in infants at high risk of BPD and with established BPD, according to the contents of Table 1 and Table 2, respectively.

In the following subsections, specific adaptations of both parenteral and enteral nutrition for infants at high risk of BPD and with established BPD are addressed.

### 7.1. Parenteral Nutrition

For healthy preterm infants, 90–120 kcal/kg/day of energy is recommended through exclusive parenteral nutrition, after the first postnatal week [75,76]. BPD infants are a special subset of preterm infants who were found to receive a lower energy intake than those without BPD in the first month of life [77]. Therefore, in infants at high risk of BPD, an energy intake of 80–100 kcal/kg/day should be provided in the first postnatal week, and 120–150 kcal/kg/day between the second and fourth postnatal weeks [44,78].

In healthy preterm infants, 1.5–2.0 g/kg/day of amino acids should be provided as soon as possible after birth, increasing to 3.5 g/kg/day for the first 48–72 postnatal hours [79]. 

Although the maximum recommended dose of glucose through parenteral nutrition is 12 mg/kg/min, its maximum oxidation capacity is 8.3 mg/kg/min in preterm infants (12 g/kg/day). In infants at risk of BPD or with established BPD, the administration of high doses of glucose can cause excessive carbon dioxide production [78]. 

To meet the recommended energy intake, intravenous lipids are a good source of energy and essential fatty acids [80]. In addition, the administration of lipids to critically ill patients decreases the *de novo* lipogenesis from glucose and carbon dioxide production [80]. In healthy preterm infants, it is recommended to start at 1.0–2.0 g/kg/day within the first 24 postnatal hours, with an increase of 0.5–1.0 g/kg/day up to a maximum of 4.0 g/kg/day from 72–96 postnatal hours [80]. A minimum of 1.0 g/kg/day of parenteral lipids is recommended to avoid a deficit in essential fatty acids [80]. The current 20% intravenous fat emulsions (IVFEs) containing soybean oil, medium-chain triglycerides (MCT), and/or fish oil are a good option for the more complete supply of essential fatty acids to preterm infants [81,82]. In addition, new-generation IVFEs, particularly those rich in MCT and fish oil, were reported to reduce lipid peroxidation, oxidative stress, and inflammatory response due to their high n-3 polyunsaturated fatty acid content and low content of n-6 fatty acids [83]. Theoretically, the composition of new-generation IVFEs could alleviate inflammation in preterm infants receiving parenteral nutrition and reduce morbidities such as BPD [82]. However, a systematic review and meta-analysis that included 22 studies and involved 3781 preterm infants, determining the associations of different IVFEs with the occurrence of BPD, found no evidence that the new-generation IVFEs could prevent BPD, including those containing fish oil [81]. 

In a prospective study examining 135 preterm infants weighing less than 1250 g, those with BPD had a suboptimal bone growth and a higher incidence of metabolic bone disease [77]. This was due to a nutritional deficit in calcium and phosphorus rather than problems with vitamin D metabolism [77]. On the other hand, chronic use of diuretics to reduce fluid overload in BPD increased the urinary loss of calcium [84]. Hydrochlorothiazide is preferred due to its calcium-sparing effect compared with furosemide [84,85]. Recently, doses of calcium and phosphate provided through PN were reviewed and much higher doses were recommended than previously: in the first postnatal week Ca 32–80 mg/kg/day and P 31–62 mg/kg/day; subsequently, Ca 100–140 mg/kg/day and P 77–108 mg/kg/day, with a Ca/P ratio of 1.3 (mass) or 1 (molar) [76,86]. These very high doses are difficult to achieve in infants with BPD due to fluid restriction and the inherent risk of calcium phosphate precipitation in parenteral nutrition solutions [87,88]. During the first postnatal days, it seems prudent not to exceed concentrations of Ca and P of 68 mg/dL and 52.7 mg/dL, respectively [89]. 

Adequate doses of vitamins should be provided through parenteral nutrition, since preterm infants are especially prone to vitamin deficits due to their limited stores and rapid growth rate [76,90]. As mentioned before, a deficit of some vitamins in preterm infants was associated with the risk of BPD [33,51,62].

Trace elements such as zinc, copper, manganese, chromium, selenium, and molybdenum are also currently recommended in neonatal long-term parenteral nutrition, including in infants with BPD, since some of them are important co-factors of antioxidant enzymes [70,76,91]. 

### 7.2. Enteral Nutrition While in the Hospital

#### 7.2.1. Enteral Nutrition Recommendations

A fluid intake of 135 mL/kg/day is considered the minimum enteral volume to supply sufficient energy and nutrients in healthy preterm infants [92], and this is the maximum fluid intake tolerated by many infants with established BPD [32,34].

It is estimated that infants with BPD require 15–25% more energy than those without BPD [57]. An energy intake in the range of 120–150 kcal/kg/day is needed in BPD infants [44], although a minimum of 140 kcal/kg/day may be necessary during active periods of the disease [57,93]. Providing such high-energy intake in low volumes of feeds remains a challenge and requires concentrating energy and macronutrients [20,23]. 

In a cohort study assessing the nutritional supply in very preterm infants, those who developed BPD received an energy intake and energy/protein ratio below that recommended for growth [20]. In line with these results, in a retrospective case–control study, it was found that extremely preterm infants with BPD received lower energy and fluid intake during the first month of life, and a lower four-week averaged daily energy intake was an independent predictor for BPD [23]. 

From the scarce randomized trials demonstrating the efficacy of nutritional interventions in the prevention or treatment of BPD, the interventional cohort study by Gianni et al. [44] showed that in very preterm infants with BPD, a greater protein/energy supply allowed for faster weight gain than in a historical cohort. 

As few studies have evaluated the protein needs of infants with BPD, it may be appropriate to assume that these are comparable to the needs of preterm infants without BPD. The ESPGHAN recommends enteral protein intakes of 3.5–4.0 g/kg/day for infants weighing 1000–1800 g and 4.0–4.5 g/kg/day for those with less than 1000 g [92].

Fat is a good source of nonprotein energy when it is necessary to provide high energy intake in a low volume of feedings [93,94]. For preterm infants with BPD, fat provides essential fatty acids and lipid soluble vitamins, and its oxidation produces less carbon dioxide [92]. An enteral intake of 4.8–6.6 g/kg/day of lipids, including 385–1540 mg/kg/day of LA and more than 50 mg/kg/day of α-linolenic acid, is recommended for healthy, growing preterm infants [92]. 

Long-term use of steroids and loop diuretics and limited mineral intake due to fluid restriction are recognized additional risk factors for metabolic bone disease in infants with BPD [95]. However, the ability of VLBW infants with early BPD to grow and accrete calcium was reported to be similar to that of those without early BPD [96]. Providing in a low volume of feedings the high intakes of Ca (120–140 mg/kg/day) and P (60–90 mg/kg/day) recommended by the European Society for Paediatric Gastroenterology Hepatology and Nutrition (ESPGHAN) [92] or the even higher intakes of Ca (150–220 mg/kg/day) and P (75–140 mg/kg/day) recommended by the American Academy of Pediatrics is challenging [97]. For healthy preterm infants, the recommended intake of magnesium is 8–15 mg/kg/day [92] and that of vitamin D varies from 400–1000 IU/day [98] to 800–1000 IU/day [92].

In a single-center study including 243 infants of less than 32 weeks’ gestation, the incidence of anemia was higher in BPD infants than non-BPD infants, and the number of transfusions was a significant risk factor for BPD [99]. Therefore, prevention and treatment of early anemia are necessary, and reducing the number of transfusions may reduce the incidence of BPD in preterm infants [99]. For preterm infants, iron supplementation at 2–4 mg/kg/day from 4–8 postnatal weeks to 12–15 months of life has been suggested [100,101].

Prolonged diuretics use to manage fluid balance in infants with BPD may cause hyponatremia, requiring sodium supplementation to maintain the sodium stores necessary for optimal growth [12]. In a retrospective study including 340 very preterm infants, it was found that severe late-onset hyponatremia (<130 mEq/L) affected the development of BPD (OR 2.95; 95% CI 1.06–8.24), despite not affecting growth beyond the neonatal period [102].

#### 7.2.2. Type of Feedings

##### Human Milk

Mother’s own milk (MOM) is the first choice for feeding healthy preterm infants due to its unique nutritional and biological properties. When MOM is not available, pasteurized donor human milk (DHM) is the second choice if human bank milk is available [19,101]. 

A systematic review and meta-analysis gathering 22 observational and interventional studies and involving 8661 preterm infants evaluated the effects of human milk (HM) on the risk of BPD and concluded that both exclusive and partial HM feeding were associated with lower risk of BPD, although the quality of evidence was low [103]. It is well known that HM has nutritional and bioactive components, including cytokines, antioxidants, lactoferrin, lysozymes, secretory immunoglobulin A, and growth factors that counteract oxidative stress and inflammation [104,105]. However, it is not yet known which components can exert a protective effect against BPD.

Some systematic reviews and meta-analyses have assessed specifically the association between feeding with MOM, DHM, or formula and the risk of BPD. Villamor-Martínez et al. [106], in a systematic review and meta-analysis of 15 studies involving 4984 very preterm infants (1416 BPD cases), assessed whether MOM could protect from BPD. They concluded that MOM could reduce the incidence of BPD (RR 0.74; 95% CI 0.57–0.96, five studies) when used as an exclusive diet. These results should be interpreted with caution since the analysis was not adequately powered to detect changes in BPD rates and adjust for confounders [106]. Based on another systematic review and a meta-analysis of 18 studies including very preterm infants, the same authors concluded that DHM also protects against BPD [107]. Specifically, the supplementation with DHM reduced BPD (RR 0.78; 95% CI 0.67–0.90, eight studies), as it happened with an exclusive HM diet compared with a preterm formula and/or a bovine milk-based fortifier (RR 0.80; 95% CI 0.68–0.95, three studies) [107]. Feeding raw MOM compared with pasteurized MOM protected against BPD (RR 0.77; 95% CI 0.62–0.96, two studies) [106]. In contrast, another systematic review and meta-analysis examining the effect of HM on morbidity of VLBW infants did not find conclusive evidence for the effect of exclusive HM feeding on BPD versus exclusive preterm formula feeding [108]. 

To prevent nutritional insufficiencies of HM while taking advantage of its biological properties, HM multinutrient fortifiers were conceived for HM-fed preterm infants [109]. Using the standard fortification method, a fixed dose of fortifier is added to HM, not taking into account the great variability in its nutritional composition [110]. In consequence, poor nutritional support due to the overestimation of the energy and macronutrient content of HM associated with suboptimal growth and adiposity were reported in growing preterm infants [111,112]. To overcome the problems inherent in standard fortification, alternative individualized HM fortification methods have been proposed, particularly adjusted fortification and target fortification [113]. In adjusted fortification, a modular protein supplement is added to fortified HM, oriented by blood urea nitrogen (BUN) as an indicator of the infant metabolic response [114]. The target fortification is based on the regular measurement of the energy and macronutrient contents of HM, in order to customize the target energy and macronutrients to each infant [115]. To achieve the desirable nutrient targets, modular supplements of protein, carbohydrate, and fat may further be added to the fortified HM [101,114,115,116]. In an interventional cohort study, the target HM fortification resulted in improved weight gain velocity in BPD infants [44]. In order to comply with the ESPGHAN enteral nutrition recommendations for preterm infants [92], the target fortification seems an attractive and precise method to customize energy and macronutrient in HM-fed infants with BPD subjected to fluid restriction. However, the target HM fortification requires an HM analyzer that is not available in most neonatal units [117]. 

Supplementation with multivitamin and iron is recommended for HM-fed infants, even when fortified HM is used [27]. In a retrospective cohort study in ELBW infants, zinc supplementation improved growth in HM-fed infants with BPD [118].

##### Preterm Formulas

When HM is insufficient or unavailable, healthy preterm infants should be fed preterm formulas while in the hospital [19,119]. These enriched formulas containing high energy and protein densities are of particular use in fluid-restricted infants with BPD [120].

Preterm formulas are advantageous since their high nutrient density and sources of macronutrients match the nutritional needs and digestive capacity of preterm infants, compared with formulas conceived for term infants [19,121]. In particular, preterm formulas contain extra energy, protein, calcium, and phosphorus, and the fat source is a blend of vegetable oils containing long-chain triglycerides and MCT [121]. In preterm infants, MCT have the theoretical advantage of allowing for a better capacity for lingual and gastric lipases to hydrolyze fatty acids of medium carbon chain length, not requiring a large bile salt pool for their absorption, and being potentially better for energy production than longer-chain fatty acids [21,122]. 

In some BPD infants with worse pulmonary function requiring greater fluid restriction, some authors have proposed increasing the energy and macronutrient intake by adding modular supplements of protein, carbohydrate, and fat to enriched formulas [123]. This formula manipulation has the inherent risks of increasing the osmolality of feedings and compromising the energy/protein ratio [124].

Infants with BPD with early signs of metabolic bone disease of prematurity should receive extra calcium, phosphorus, and vitamin D, provided by fortified HM or preterm formula [33]. 

#### 7.2.3. Timing of Initiation

For healthy preterm infants, the ESPGHAN recommends to start feeding with minimal enteral nutrition or trophic feeding, defined as the supply of nutritionally insignificant milk volumes of 12–24 mL/kg/day to maintain intestinal integrity [125].

The timing of initiation of enteral feeding may have an impact on intestinal inflammation and risk of neonatal comorbidities. In a retrospective cohort study of 133 infants born at less than 33 weeks of gestation, the effect of the timing of the first enteral feeding on the inflammatory state of the intestinal tract and risk of neonatal morbidities was evaluated [126]. Late enteral feeding, which was considered the initial enteral feeding after the third postnatal day, was associated with increased fecal IL-8 levels and a decreased IL-10:IL-8 ratio [126]. After multivariate analysis, late enteral feeding was found to be associated with a 4.5-fold increase of BPD (95% CI 1.8–11.5; *p* = 0.002) [126]. These results are consistent with a secondary analysis of a prospective multicenter cohort study, in which elevated circulating IL-8 levels were found in 606 ELBW infants who developed BPD or died [127].

#### 7.2.4. Feeding Methods

Tube feeding for a long period of time may be required in very preterm infants with DBP [27]. In these infants, several repeated negative stimuli contribute to difficulties in feeding by mouth, including prolonged orotracheal intubation, hypoxic episodes, tachypnea, altered gastrointestinal motility associated with the use of methylxanthines, and irritability related with neurological status [27]. In addition, the prevalence of gastroesophageal reflux (GER) is high in BPD infants, particularly acid reflux in ELBW infants [128,129]. Episodes of acid reflux have been implicated in the worsening of lung disease, and the occurrence and frequency of symptoms in BPD, depending on the most proximal esophageal extent of the acid reflux and acid clearance [128]. An observational cohort study monitored esophageal pH impedance in symptomatic infants of less than 33 weeks’ gestation with and without BPD [130]. Infants with BBP, compared with those without BPD, had more frequent pH-only events (median number 21 vs. 9) and a higher symptom sensitivity index for pH-only events (9% vs. 4.9%). These findings may be explained by impaired esophageal motility and a peculiar autonomic nervous system response pattern in infants with BPD [130]. Considering the increasing concerns over the safety of antiacid drugs in preterm infants, pharmacologic treatment for GER should be initiated only after the demonstration of pathological pH-MII monitoring [130]. Transpyloric feedings have been suggested to reduce episodes of apnea and bradycardia in GER [131]. However, a trial in very preterm infants with severe BPD did not demonstrate the advantage of transpyloric feedings compared with gastric feedings in relation to the frequency of hypoxemia [132]. In more severe cases of GER, gastrostomy tube placement along with fundoplication may be necessary [27]. 

Deglutition apnea during non-nutritive suckling has been evaluated in relation to swallowing and breathing in infants with low risk of BPD and at risk of developing BPD [133]. No significant differences were noted between groups in relation to the swallow–breath interaction and the likelihood of respiration incidents [133]. In addition, no effect on these parameters was found with an individualized intervention by a speech–language specialist [133].

#### 7.2.5. Tracheostomy

The decision about whether and when to place a tracheostomy in infants with severe BPD dependent on a prolonged ventilator is difficult for both families and clinicians [11]. In these cases, tracheostomy has the advantage of reducing agitation and sedative medication and allowing a better parent–child interaction [11]. 

In a retrospective cohort study including 72 very preterm infants with BPD who required tracheostomy, significant improvements in weight and length were recorded by four weeks after tracheostomy placement [134]. In particular, the median length *z*-score increased from −3.07 to −1.95, curiously associated with a significant reduction in the mean energy intake, from 108.9 to 93.8 kcal/kg/day [134]. 

### 7.3. Nutrition after Discharge

In infants with BPD, discharge should be carefully organized based on a plan decided in agreement with a multidisciplinary team, ideally consisting of a neonatologist, a neonatal nurse, a pulmonologist, and a nutritionist [12,14].

Infants with BPD often have difficulty in achieving coordinated suckle feeding, which initially limits breast feeding or the use of a bottle [135,136]. The low feeding efficiency is due to low sucking pressure and sucking frequency, short sucking duration, gagging, high respiratory rate, an excessive decrease in oxygen saturation, and long deglutition apnea [27,119]. As mentioned before, infants with BPD have a higher incidence of GER, which increases the risk of aspiration, pneumonia, apnea, and failure to thrive [119]. 

In a cross-sectional study of 4–8-year-old children who had been born prematurely and suffered BPD, it was concluded that undernutrition at the age of two years was the only factor associated with the risk of developing distension of the airway [120]. 

As BPD infants do not tolerate high fluid intake, feedings highly concentrated in energy and nutrients should be offered after discharge, using either fortified breastmilk or enriched formula [119]. Investigation is needed to clarify whether BPD infants have similar nutritional advantages when fed fortified breastmilk, as described in preterm infants without BPD [109,137].

When the growth of exclusively breastfed BPD infants is poor, postdischarge formulas may be an option [138,139], alternating with breastfeeding or in place of breastfeeding. Postdischarge formulas are enriched with energy and nutrients designed for short- to medium-term use in healthy preterm infants after discharge, and their energy and nutrient content is in between that of preterm formulas and term formulas [136]. 

If poor growth persists despite using a postdischarge formula, this may be replaced with a preterm formula, providing in the same volume (100 mL) more energy (circa 80 vs. 73 kcal), protein (circa 2.4 vs. 1.9 g), calcium (circa 140 vs. 80 mg), and phosphorus (circa 75 vs. 50 mg) [136,139]. In a trial, BPD infants fed preterm formula attained significantly greater length, bone mineral content, and lean mass at three months corrected age than those fed a term formula [122].

## 8. Monitoring

### 8.1. While in the Hospital

Anthropometry should be used to monitor the growth of BPD infants, as recommended for preterm infants under intensive care [140] (Table 3). Body weight should be measured daily and length and head circumference weekly, using correct methods and appropriate charts to interpret the measurements [140].

Accurate fluid management is crucial for infants at risk of developing BPD. Therefore, a tool sensitive enough to detect sudden weight changes will indicate inappropriate extracellular water changes rather than changes in body lean or fat mass [140,141]. Charts that do not take into account the initial weight loss in the result of physiological reduction of extracellular volume, such as the Fenton 2013 charts [142], are inappropriate for monitoring short-term postnatal weight changes [143]. A comprehensive longitudinal study provided new charts to monitor body weight [144]. Based on this study, a growth calculator that displays graphically in which percentile the current weight is plotted was developed [141]. This free-access online tool (https://www.growthcalculator.org/) (accessed on 21 April 2021) is sensitive to accurately detect weight changes, exhibiting the target weight and the deviation of the current weight.

After the initial physiological weight loss, weight gain velocity (in g/kg/day) is more sensitive for identifying changes in growth than examining the weight plotted on growth curves [144,145]. For a precise calculation of weight gain velocity, the optimum period seems to be a time interval of at least 5–7 days [146]. Body weight gain rates of 15–20 g/kg/day are considered adequate for infants born at 23–36 weeks of gestation [48]. 

Length and head circumference growth velocities provide sensitive measurements of linear and head growth: rates of 0.9–1.1 cm/week and 0.9–1.0 cm/week, respectively, are adequate for preterm infants, particularly for 23–30 weeks PMA [140,147]. 

Some biochemical markers are useful in the assessment of nutritional status and should complement the anthropometric assessment (Table 3). In clinically stable preterm infants, blood urea nitrogen (BUN) is useful to monitor the adequacy of enteral protein intake, and may guide the use of HM fortifiers and the addition of modular protein when necessary [113,139]. A BUN value lower than 1.6 mmol/L (4.48 mg/dL) indicates insufficient protein intake [140,148]. In preterm infants, complete blood count with reticulocyte count and serum ferritin levels should be regularly measured to monitor iron status [27,140]. Low phosphorus and high alkaline phosphate serum levels are reasonable early indicators of metabolic bone disease of prematurity [139,149]. Regular measurements of serum electrolytes are needed if diuretics are used [12,27]. In case of prolonged use of diuretics, renal ultrasound is recommended for nephrocalcinosis screening [12].

### 8.2. After Discharge

Regular outpatient follow-up with a pediatrician and subspecialists is recommended, based on anthropometry and biochemical markers [12] (Table 4). 

Body weight, length, and head circumference should be evaluated on a regular basis [12]. The longitudinal Intergrowth-21st standards are more appropriate to interpret body weight, length, and head circumference measurements up to 64 weeks PMA, for infants born at more than 26 weeks and less than 37 weeks of gestation [139,150]. 

Particularly in infants fed unfortified breast milk, surveillance of iron deficiency anemia, protein malnutrition, and metabolic bone disease is recommended. Thus, complete blood count and serum levels of ferritin, BUN, calcium, phosphorus, magnesium, and alkaline phosphate should be measured on a regular basis [12,27]. Regular monitoring of serum electrolytes and trace minerals is recommended if the infant remains on diuretics [12,27].

Serum levels of specific vitamins and trace elements, including vitamin A, 25-hydroxy vitamin D, zinc, and selenium, should be determined if deficiency is suspected [27].

## 9. Conclusions

Nutrition plays a crucial role in lung growth and repair in infants with BPD. When prescribing early nutritional support to preterm infants, protection of the lungs encompasses avoidance of fluid overload. Ideally, these infants should receive a fluid intake not exceeding 135–150 mL/kg/day. Providing an ideal energy intake of 120–150 kcal/kg/day in the presence of this fluid restriction remains a challenge and is the main reason for poor growth in DBP infants.

An adequate nutritional approach for preterm infants implies early aggressive parenteral nutrition while initiating trophic feeding and progressing to nutritive enteral nutrition as soon as possible. To provide high energy and nutrient intakes in a low volume of feedings, multinutrient fortification of the mother’s own milk or of donor milk is necessary. When HM is insufficient, enriched formulas with a high density of energy and nutrients are preferred.

Specialized nutritional techniques may be used to overcome gastroesophageal reflux and poor coordination of suckling, which are common in infants with DBP. Tracheostomy may result in nutritional advantages in infants requiring prolonged invasive ventilation. Planning nutrition support after discharge requires a multidisciplinary approach for the multiple clinical problems that these infants face. To monitor the nutritional intervention, anthropometry and appropriate growth charts and specific biochemical markers should be used.

## Figures and Tables

**Table 1:** Preventive nutritional approach in infants at high risk of BPD.

Intervention		Reference
Avoid excessive fluid intake	− In the first postnatal day: 80–100 mL/kg/day − After the first postnatal week: 135–150 mL/kg/day	[32,33,34]
Provide adequate incubator humidity	− In the first postnatal week: 60–70%	[37]
Maintain adequate temperature	− Abdominal skin: 36.0–36.5 °C − Inspired air temperature (hood, CPAP, or ventilator): 34.0–41.0 °C, relative humidity of 100%	[38,39]
Optimize early parenteral energy intake	− In the first postnatal week: 80–100 kcal/kg/day − After the first postnatal week: 120–150 kcal/kg/day	[44,78]
Optimize early parenteral amino acid intake	− Start with 1.5–2 g/kg/day after birth − Increase to 3.5 g/kg/day from the first 48–72 postnatal hours	[79]
Optimize early parenteral fat intake	− Start with 1.0–2.0 g/kg/day within the first postnatal day − Increase by 0.5–1.0 g/kg/day up to a maximum of 4.0 g/kg/day at 72–96 postnatal hours	[80]
Provide adequate intravenous glucose	− Limit the rate to 12 mg/kg/min (ideal limit: 8.3 mg/kg/min)	[33]
Optimize early parenteral calcium and phosphorus intake	− In the first postnatal week: parenteral Ca 32–80 mg/kg/day and P 31–62 mg/kg/day − After the first postnatal week: parenteral Ca 100–140 mg/kg/day and P 77–108 mg/kg/day − Parenteral Ca/P ratio: 1.3 (mass) or 1 (molar)	[86]
Provide adequate intravenous lipid soluble vitamins	− Vitamin A (retinol) 227–455 µg/kg/day or 700–1500 IU/kg/day − Vitamin E (α-tocopherol) 2.8–3.5 IU/kg/day	[90]
Provide adequate intravenous trace elements	− Particularly zinc 400–500 μg/kg/day	[91]
Initiate early enteral feeding	− Initiate minimal enteral feeding (12–24 mL/kg/day) prior to 3rd postnatal day − Use preferably mother’s own milk or donor human milk as second choice	[101,106,125]

**Table 2:** Nutritional management in infants with established BPD, either in the hospital or after discharge.

Intervention		Reference
Fluid restriction	Less than 150 mL/kg/day Ideally, up to 135 mL/kg/day	[32,33,34]
Optimize enteral energy intake	Ideally, 120–150 kcal/kg/day	[33,44]
Optimize enteral protein intake	− <1000 g body weight: 4.0–4.5 g/kg/day − 1000–1800 g body weight: 3.5–4.0 g/kg/day	[92]
Optimize enteral lipid intake	− Total lipid intake 4.8–6.6 g/kg/day − Arachidonic acid 12–30 mg/kg/day − Docosahexaenoic acid 18–42 mg/kg/day	[92]
Optimize enteral calcium and phosphate intake	− Ca 120–140 mg/kg/day *; 150–220 mg/kg/day ** − P 60–90 mg/kg/day *; 75–140 mg/kg/day ** − Ca/P ratio: 2 (mass) *	[92] [97]
Optimize sodium intake if diuretics are used	− Provide sodium supplement to maintain serum Na >135 mEq/L	[12]
Optimize enteral vitamin A intake	400–1000 µg/kg/day or 1320–3300 IU/kg/day	[92]
Optimize enteral vitamin E (α-tocopherol) intake	2.2–11 mg/kg/day	[92]
Supplemental iron	4 mg/kg/day, from 4–8 postnatal weeks up to 12 months of life	[33]

Note: * [92]; ** [97].

**Table 3:** In-hospital monitoring of infants with BPD.

Parameter		Reference
Body weight (daily)	− Body weight change: online calculator (https://www.growthcalculator.org/) (accessed on 21 April 2021) − Weight gain velocity; ideally 15–20 g/kg/day	[141] [146]
Body length (weekly)	Body length velocity: 0.9–1.1 cm/week	[147]
Head circumference (weekly)	Head circumference velocity: 0.9–1.0 cm/week	[147]
Monitoring iron status	Complete blood count with reticulocyte count, and serum ferritin levels	[140]
Monitoring protein nutrition	Blood urea nitrogen (BUN)	[113]
Monitoring early metabolic bone disease	Serum phosphorus and alkaline phosphate levels	[149]
Monitoring electrolyte balance (diuretics use)	Serum electrolytes	[27]

**Table 4:** Monitoring infants with BPD after discharge.

Parameter		Reference
Body weight, length, and head circumference	Intergrowth-21st standards: monitoring up to 64 weeks postmenstrual age, for infants born at >26 and <37 weeks of gestation	[150]
Monitoring iron status	Complete blood count with reticulocyte count, and serum ferritin levels	[140]
Monitoring protein nutrition	Blood urea nitrogen (BUN)	[113]
Monitoring metabolic bone disease	Serum phosphorus and alkaline phosphate levels	[149]
Monitoring electrolyte balance (if diuretic use)	Serum electrolytes	[27]
Monitoring vitamins and trace elements (if deficiency suspicion)	Serum levels of vitamin A, 25-hydroxy vitamin D, zinc, and selenium	[27]

## Data Availability

Not applicable.

## Abbreviations

BPD	bronchopulmonary dysplasia
BUN	blood urea nitrogen
DHA	docosahexaenoic acid
DHM	donor human milk
ELBW	extremely low birth weight
ESPGHAN	European Society for Paediatric Gastroenterology Hepatology and Nutrition
GER	gastroesophageal reflux
HM	human milk
IVFEs	intravenous fat emulsions
LA	linoleic acid
MCT	medium-chain triglycerides
MOM	mother’s own milk
PMA	postmenstrual age
VLBW	very low birth weight

## References

[1] Bancalari E., Jain D. (2019). Bronchopulmonary Dysplasia: 50 Years after the Original Description. Neonatology.

[2] Northway W.H., Rosan R.C., Porter D.Y. (1967). Pulmonary Disease Following Respirator Therapy of Hyaline-Membrane Disease. N. Engl. J. Med..

[3] Jobe A.H. (2011). The new bronchopulmonary dysplasia. Curr. Opin. Pediatr..

[4] Jobe A.H., Bancalari E. (2001). Bronchopulmonary Dysplasia. Am. J. Respir. Crit. Care Med..

[5] Higgins R.D., Jobe A.H., Koso-Thomas M., Bancalari E., Viscardi R.M., Hartert T.V., Ryan R., Kallapur S.G., Steinhorn R.H., Konduri G.G. (2018). Bronchopulmonary Dysplasia: Executive Summary of a Workshop. J. Pediatr..

[6] Stoll B.J., Hansen N.I., Bell E.F., Shankaran S., Laptook A.R., Walsh M.C., Hale E.C., Newman N.S., Schibler K., Carlo W.A. (2010). Eunice Kennedy Shriver National Institute of Child Health and Human Development Neonatal Research Network. Neonatal outcomes of extremely preterm infants from the NICHD Neonatal Research Network. Pediatrics.

[7] Guimarães H., Rocha G., Vasconcellos G., Proença E., Carreira M., Sossai M., Morais B., Martins I., Rodrigues T., Severo M. (2010). Bronchopulmonary dysplasia: Clinical practices in five Portuguese neonatal intensive care units. Rev. Port. Pneumol..

[8] Shahzad T., Radajewski S., Chao C.-M., Bellusci S., Ehrhardt H. (2016). Pathogenesis of bronchopulmonary dysplasia: When inflammation meets organ development. Mol. Cell. Pediatr..

[9] Lignelli E., Palumbo F., Myti D., Morty R.E. (2019). Recent advances in our understanding of the mechanisms of lung alveolarization and bronchopulmonary dysplasia. Am. J. Physiol. Cell. Mol. Physiol..

[10] Álvarez-Fuente M., Moreno L., Mitchell J.A., Reiss I.K., Lopez P., Elorza D., Duijts L., Avila-Alvarez A., Arruza L., Orellana M.R. (2018). Preventing bronchopulmonary dysplasia: New tools for an old challenge. Pediatr. Res..

[11] Muehlbacher T., Bassler D., Bryant M. (2021). Evidence for the Management of Bronchopulmonary Dysplasia in Very Preterm Infants. Children.

[12] Poindexter B.B., Martin C.R. (2015). Impact of Nutrition on Bronchopulmonary Dysplasia. Clin. Perinatol..

[13] Hwang J.S., Rehan V.K. (2018). Recent Advances in Bronchopulmonary Dysplasia: Pathophysiology, Prevention, and Treatment. Lung.

[14] Bose C., Van Marter L.J., Laughon M., O’Shea T.M., Allred E.N., Karna P., Ehrenkranz R.A., Boggess K., Leviton A. (2009). For the Extremely Low Gestational Age Newborn Study Investigators Fetal Growth Restriction and Chronic Lung Disease Among Infants Born Before the 28th Week of Gestation. Am. Acad. Pediatr..

[15] Soudée S., Vuillemin L., Alberti C., Mohamed D., Becquet O., Farnoux C., Biran V., Baud O. (2014). Fetal Growth Restriction Is Worse than Extreme Prematurity for the Developing Lung. Neonatology.

[16] Klinger G., Sokolover N., Boyko V., Sirota L., Lerner-Geva L., Reichman B. (2013). Perinatal risk factors for bronchopulmonary dysplasia in a national cohort of very-low-birthweight infants. Am. J. Obstet. Gynecol..

[17] Reiss I., Landmann E., Heckmann M., Misselwitz B., Gortner L. (2003). Increased risk of bronchopulmonary dysplasia and increased mortality in very preterm infants being small for gestational age. Arch. Gynecol. Obstet..

[18] Rocha G., De Lima F.F., Machado A.P., Guimarães H., Proença E., Carvalho C., Martins L., Martins T., Freitas A., Dias C. (2020). Small for gestational age very preterm infants present a higher risk of developing bronchopulmonary dysplasia. J. Neonatal-Perinatal Med..

[19] Groh-Wargo S., Sapsford A. (2009). Enteral Nutrition Support of the Preterm Infant in the Neonatal Intensive Care Unit. Nutr. Clin. Pract..

[20] Milanesi B.G., Lima P.A., Villela L.D., Martins A.S., Gomes-Junior S.C.S., Moreira M.E.L., Méio M.D.B.B. (2021). Assessment of early nutritional intake in preterm infants with bronchopulmonary dysplasia: A cohort study. Eur. J. Nucl. Med. Mol. Imaging.

[21] Al-Jebawi Y., Agarwal N., Wargo S.G., Shekhawat P., Mhanna M. (2020). Low caloric intake and high fluid intake during the first week of life are associated with the severity of bronchopulmonary dysplasia in extremely low birth weight infants. J. Neonatal-Perinatal Med..

[22] Johnson Y.R., Brozanski B., Farrow K.N., Zaniletti I., Padula M.A., Asselin J.M., Durand D.J., Short B.L., Pallotto E.K., Dykes F.D. (2013). Postnatal Weight Gain in Preterm Infants with Severe Bronchopulmonary Dysplasia. Am. J. Perinatol..

[23] Malikiwi A.I., Lee Y.-M., Davies-Tuck M., Wong F.Y. (2019). Postnatal nutritional deficit is an independent predictor of bronchopulmonary dysplasia among extremely premature infants born at or less than 28 weeks gestation. Early Hum. Dev..

[24] Marques P.C., Rocha G., Flor-De-Lima F., Guimarães H. (2018). Extrauterine growth restriction at discharge in very low birth weight infants: A retrospective study in a level III neonatal intensive care unit. Minerva Pediatr..

[25] Bauer K., Versmold H. (1989). Postnatal weight loss in preterm neonates less than 1500 g is due to isotonic dehydration of the extracellular volume. Acta Paediatr. Scand Suppl..

[26] Stephens B.E., Gargus R.A., Walden R.V., Mance M., Nye J., McKinley L., Tucker R., Vohr B.R. (2008). Fluid regimens in the first week of life may increase risk of patent ductus arteriosus in extremely low birth weight infants. J. Perinatol..

[27] Biniwale M.A., Ehrenkranz R.A. (2006). The Role of Nutrition in the Prevention and Management of Bronchopulmonary Dysplasia. Semin. Perinatol..

[28] Wemhöner A., Ortner D., Tschirch E., Strasak A., Rüdiger M. (2011). Nutrition of preterm infants in relation to bronchopulmonary dysplasia. BMC Pulm. Med..

[29] Oh W., Poindexter B.B., Perritt R., Lemons J.A., Bauer C.R., Ehrenkranz R.A., Stoll B.J., Poole K., Wright L.L. (2005). Neonatal Research Network. Association between Fluid Intake and Weight Loss during the First Ten Days of Life and Risk of Bronchopulmonary Dysplasia in Extremely Low Birth Weight Infants. J. Pediatr..

[30] Bertrand O.B.J., Battisti J.-M.B.O. (2012). Lung Compliance and Airways Resistance in Healthy Neonates. Pediatr. Ther..

[31] Guo M.M.-H., Chung C.-H., Chen F.-S., Chen C.-C., Huang H.-C., Chung M.-Y. (2014). Severe Bronchopulmonary Dysplasia is Associated with Higher Fluid Intake in Very Low-Birth-Weight Infants: A Retrospective Study. Am. J. Perinatol..

[32] Bell E.F., Acarregui M.J. (2014). Restricted versus liberal water intake for preventing morbidity and mortality in preterm infants. Cochrane Database Syst. Rev..

[33] Zhang R., Lin X.Z., Chang Y.M., Liu X.H., Tong X.M., Ding G.F., Nutritional Committee of Neonatology Branch of Chinese Medical Doctor Association, Editorial Committee of Chinese Journal of Contemporary Pediatrics (2020). Expert consensus on nutritional management of preterm infants with bronchopulmonary dysplasia. Chin. J. Contemp. Paediatr..

[34] Barrington K.J., Fortin-Pellerin E., Pennaforte T. (2017). Fluid restriction for treatment of preterm infants with chronic lung disease. Cochrane Database Syst. Rev..

[35] Principi N., Di Pietro G.M., Esposito S. (2018). Bronchopulmonary dysplasia: Clinical aspects and preventive and therapeutic strategies. J. Transl. Med..

[36] Sinclair J.C. (2002). Servo-control for maintaining abdominal skin temperature at 36C in low birth weight infants. Cochrane Database Syst. Rev..

[37] Glass L., Valdez A. (2020). Preterm Infant Incubator Humidity Levels: A systematic review. Adv. Neonatal Care.

[38] Sinclair L., Crisp J., Sinn J. (2009). Variability in incubator humidity practices in the management of preterm infants. J. Paediatr. Child Health.

[39] Restrepo R.D., Walsh B.K., Toussaint M., Guillet M.-C., Paternotte S., Soudon P., Haan J. (2012). Humidification During Invasive and Noninvasive Mechanical Ventilation: 2012. Respir. Care.

[40] Stokowski L.A. (2006). Fundamentals of phototherapy for neonatal jaundice. Adv. Neonatal Care.

[41] Uberos J., Lardón-Fernández M., Machado-Casas I., Molina-Oya M., Narbona-López E. (2016). Nutrition in extremely low birth weight infants: Impact on bronchopulmonary dysplasia. Minerva Paediatr..

[42] Klevebro S., Westin V., Sjöström E.S., Norman M., Domellöf M., Bonamy A.-K.E., Hallberg B. (2019). Early energy and protein intakes and associations with growth, BPD, and ROP in extremely preterm infants. Clin. Nutr..

[43] Theile A.R., Radmacher P.G., Anschutz T.W., Davis D.W., Adamkin D.H. (2011). Nutritional strategies and growth in extremely low birth weight infants with bronchopulmonary dysplasia over the past 10 years. J. Perinatol..

[44] Gianni M.L., Roggero P., Colnaghi M.R., Piemontese P., Amato O., Orsi A., Morlacchi L., Mosca F. (2014). The role of nutrition in promoting growth in pre-term infants with bronchopulmonary dysplasia: A prospective non-randomised interventional cohort study. BMC Pediatr..

[45] Allen J., Zwerdling R., Ehrenkranz R., Gaultier C., Geggel R., Greenough A., Kleinman R., Klijanowicz A., Martinez F., Ozdemir A. (2003). Statement on the Care of the Child with Chronic Lung Disease of Infancy and Childhood. Am. J. Respir. Crit. Care Med..

[46] Pereira G.R., Baumgart S., Bennett M.J., Stallings V.A., Georgieff M.K., Hamosh M., Ellis L. (1994). Use of high-fat formula for premature infants with bronchopulmonary dysplasia: Metabolic, pulmonary, and nutritional studies. J. Pediatr..

[47] Kashyap S., Towers H.M., Sahni R., Ohira-Kist K., Abildskov K., Schulze K.F. (2001). Effects of quality of energy on substrate oxidation in enterally fed, low-birth-weight infants. Am. J. Clin. Nutr..

[48] Fenton T.R., Anderson D., Groh-Wargo S., Hoyos A., Ehrenkranz R.A., Senterre T. (2018). An Attempt to Standardize the Calculation of Growth Velocity of Preterm Infants—Evaluation of Practical Bedside Methods. J. Pediatr..

[49] Fink N.H., Collins C.T., Gibson R., Makrides M., Penttila I.A. (2016). Targeting inflammation in the preterm infant: The role of the omega-3 fatty acid docosahexaenoic acid. J. Nutr. Intermed. Metab..

[50] Manley B.J., Makrides M., Collins C.T., McPhee A.J., Gibson R.A., Ryan P., Sullivan T.R., Davis P.G. (2011). For the DINO Steering Committee High-Dose Docosahexaenoic Acid Supplementation of Preterm Infants: Respiratory and Allergy Outcomes. Am. Acad. Pediatr..

[51] Falciglia H.S., Johnson J.R., Sullivan J., Hall C.F., Miller J.D., Riechmann G.C., Falciglia G.A. (2003). Role of Antioxidant Nutrients and Lipid Peroxidation in Premature Infants with Respiratory Distress Syndrome and Bronchopulmonary Dysplasia. Am. J. Perinatol..

[52] Martin C.R., DaSilva D.A., Cluette-Brown J.E., DiMonda C., Hamill A., Bhutta A.Q., Coronel E., Wilschanski M., Stephens A.J., Driscoll D.F. (2011). Decreased Postnatal Docosahexaenoic and Arachidonic Acid Blood Levels in Premature Infants are Associated with Neonatal Morbidities. J. Pediatr..

[53] Marc I., Piedboeuf B., Lacaze-Masmonteil T., Fraser W., Mâsse B., Mohamed I., Qureshi M., Afifi J., Lemyre B., Caouette G. (2020). Effect of maternal docosahexaenoic acid supplementation on bronchopulmonary dysplasia-free survival in breastfed preterm infants: A randomized clinical trial. JAMA.

[54] Collins C.T., Makrides M., McPhee A.J., Sullivan T., Davis P.G., Thio M., Simmer K., Rajadurai V.S., Travadi J., Berry M.J. (2017). Docosahexaenoic Acid and Bronchopulmonary Dysplasia in Preterm Infants. N. Engl. J. Med..

[55] Wang Q., Zhou B., Cui Q., Chen C. (2019). Omega-3 Long-chain Polyunsaturated Fatty Acids for Bronchopulmonary Dysplasia: A Meta-analysis. J. Pediatr..

[56] Tanaka K., Tanaka S., Shah N., Ota E., Namba F. (2020). Docosahexaenoic acid and bronchopulmonary dysplasia in preterm infants: A systematic review and meta-analysis. J. Matern. Neonatal Med..

[57] Dani C., Poggi C. (2012). Nutrition and bronchopulmonary dysplasia. J. Matern. Neonatal Med..

[58] Moe-Byrne T., E Wagner J.V., McGuire W., Moe-Byrne T. (2012). Glutamine supplementation to prevent morbidity and mortality in preterm infants. Cochrane Database Syst. Rev..

[59] Ahola T., Lapatto R., O Raivio K., Selander B., Stigson L., Jonsson B., Jonsbo F., Esberg G., Stövring S., Kjartansson S. (2003). N-acetylcysteine does not prevent bronchopulmonary dysplasia in immature infants: A randomized controlled trial. J. Pediatr..

[60] Mactier H. (2005). Vitamin A and preterm infants: What we know, what we don’t know, and what we need to know. Arch. Dis. Child. Fetal Neonatal Ed..

[61] Mank E., Naninck E.F.G., Limpens J., Van Toledo L., Van Goudoever J.B., Akker C.H.P.V.D. (2020). Enteral Bioactive Factor Supplementation in Preterm Infants: A Systematic Review. Nutrients.

[62] Tyson J.E., Wright L.L., Oh W., Kennedy K.A., Mele L., Ehrenkranz R.A., Stoll B.J., Lemons J.A., Stevenson D.K., Bauer C.R. (1999). Vitamin A Supplementation for Extremely-Low-Birth-Weight Infants. N. Engl. J. Med..

[63] Ambalavanan N., Tyson J.E., Kennedy K.A., Hansen N.I., Vohr B.R., Wright L.L., Carlo W.A. (2005). National Institute of Child Health and Human Development Neonatal Research Network. Vitamin A supplementation for extremely low birth weight infants: Outcome at 18 to 22 months. J. Pediatr..

[64] Pearson E., Bose C., Snidow T., Ransom L., Young T., Bose G., Stiles A. (1992). Trial of vitamin A supplementation in very low birth weight infants at risk for bronchopulmonary dysplasia. J. Pediatr..

[65] Kennedy K.A., Stoll B.J., Ehrenkranz R.A., Oh W., Wright L.L., Stevenson D.K., Lemons J.A., Sowell A., Mele L., Tyson J.E. (1997). The NICHD Neonatal Research Network. Vitamin A to prevent bronchopulmonary dysplasia in very-low-birth-weight infants: Has the dose been too low?. Early Hum. Dev..

[66] Araki S., Kato S., Namba F., Ota E. (2018). Vitamin A to prevent bronchopulmonary dysplasia in extremely low birth weight infants: A systematic review and meta-analysis. PLoS ONE.

[67] Lee G.Y., Han S.N. (2018). The Role of Vitamin E in Immunity. Nutrients.

[68] Ehrenkranz R.A., Ablow R.C., Warshaw J.B. (1982). Effect of vitamin E on the development of oxygen-induced lung injury in neonates. Ann. N. Y. Acad. Sci..

[69] Ohlsson A. (2003). Inositol for respiratory distress syndrome in preterm infants. Cochrane Database Syst. Rev..

[70] Bocca B., Ciccarelli S., Agostino R., Alimonti A. (2017). Trace elements, oxidative status and antioxidant capacity as biomarkers in very low birth weight infants. Environ. Res..

[71] Loui A., Raab A., Maier R.F., Brätter P., Obladen M. (2010). Trace elements and antioxidant enzymes in extremely low birthweight infants. J. Trace Elements Med. Biol..

[72] Vázquez-Gomis R., Bosch-Gimenez V., Juste-Ruiz M., Vázquez-Gomis C., Izquierdo-Fos I., Pastor-Rosado J. (2019). Zinc concentration in preterm newborns at term age, a prospective observational study. BMJ Paediatr. Open.

[73] Terrin G., Canani R.B., Passariello A., Messina F., Conti M.G., Caoci S., Smaldore A., Bertino E., De Curtis M. (2013). Zinc supplementation reduces morbidity and mortality in very-low-birth-weight preterm neonates: A hospital-based randomized, placebo-controlled trial in an industrialized country. Am. J. Clin. Nutr..

[74] A Darlow B., Austin N. (2003). Selenium supplementation to prevent short-term morbidity in preterm neonates. Cochrane Database Syst. Rev..

[75] Pereira-da-Silva L., Pissarra S., Alexandrino A.M., Malheiro L., Macedo I., Cardoso M., Vieira-da-Silva P., Frutuoso S.P., Lau H., Soares T. (2019). On behalf of the Portuguese Neonatal Society Guidelines for Neonatal Parenteral Nutrition: 2019 Update by the Portuguese Neonatal Society. Part I. General Aspects, Energy, and Macronutrients. Port. J. Pediatr..

[76] Pereira-da-Silva L., Pissarra S., Alexandrino A.M., Malheiro L., Macedo I., Cardoso M., Vieira-da-Silva P., Frutuoso S.P., Lau H., Soares T. (2019). On behalf of the Portuguese Neonatal Society Guidelines for Neonatal Parenteral Nutrition: 2019 Update by the Portuguese Neonatal Society. Part II. Micronutrients, Ready-to-use Solutions and Particular Conditions. Port. J. Pediatr..

[77] Gaio P., Verlato G., Daverio M., Cavicchiolo M.E., Nardo D., Pasinato A., de Terlizzi F., Baraldi E. (2018). Incidence of metabolic bone disease in preterm infants of birth weight <1250 g and in those suffering from bronchopulmonary dysplasia. Clin. Nutr. ESPEN.

[78] Joosten K., Embleton N., Yan W., Senterre T., Braegger C., Bronsky J., Cai W., Campoy C., Carnielli V., Darmaun D. (2018). ESPGHAN/ESPEN/ESPR/CSPEN guidelines on pediatric parenteral nutrition: Energy. Clin. Nutr..

[79] Van Goudoever J.B., Carnielli V., Darmaun D., De Pipaon M.S., Braegger C., Bronsky J., Cai W., Campoy C., Decsi T., Domellöf M. (2018). ESPGHAN/ESPEN/ESPR/CSPEN guidelines on pediatric parenteral nutrition: Amino acids. Clin. Nutr..

[80] Lapillonne A., Mis N.F., Goulet O., van den Akker C.H.V.D., Wu J., Koletzko B., Braegger C., Bronsky J., Cai W., Campoy C. (2018). ESPGHAN/ESPEN/ESPR/CSPEN guidelines on pediatric parenteral nutrition: Lipids. Clin. Nutr..

[81] Fan X., Tang Y., Tang J., Chen J., Shi J., Wang H., Xia B., Qu Y., Mu D. (2020). New-generation intravenous fat emulsions and bronchopulmonary dysplasia in preterm infants: A systematic review and meta-analysis. J. Perinatol..

[82] Salama G.S.A., Kaabneh M.A., Almasaeed M.N., Alquran M.I. (2015). Intravenous Lipids for Preterm Infants: A Review. Clin. Med. Insights: Pediatr..

[83] Guthrie G., Premkumar M., Burrin D.G. (2017). Emerging Clinical Benefits of New-Generation Fat Emulsions in Preterm Neonates. Nutr. Clin. Pract..

[84] Faienza M.F., D’Amato E., Natale M.P., Grano M., Chiarito M., Brunetti G., D’Amato G. (2019). Metabolic Bone Disease of Prematurity: Diagnosis and Management. Front. Pediatr..

[85] Carlson S.J. (2004). Current Nutrition Management of Infants with Chronic Lung Disease. Nutr. Clin. Pract..

[86] Mihatsch W., Fewtrell M., Goulet O., Molgaard C., Picaud J.-C., Senterre T., Braegger C., Bronsky J., Cai W., Campoy C. (2018). ESPGHAN/ESPEN/ESPR/CSPEN guidelines on pediatric parenteral nutrition: Calcium, phosphorus and magnesium. Clin. Nutr..

[87] Mackay M., Jackson D., Eggert L., Fitzgerald K., Cash J. (2011). Practice-Based Validation of Calcium and Phosphorus Solubility Limits for Pediatric Parenteral Nutrition Solutions. Nutr. Clin. Pract..

[88] Watrobska-Swietlikowska D. (2019). Compatibility of Maximum Inorganic and Organic Calcium and Phosphate Content in Neonatal Parenteral Solutions. Sci. Rep..

[89] Mulla S., Stirling S., Cowey S., Close R., Pullan S., Howe R., Radbone L., Clarke P. (2017). Severe hypercalcaemia and hypophosphataemia with an optimised preterm parenteral nutrition formulation in two epochs of differing phosphate supplementation. Arch. Dis. Child. Fetal Neonatal Ed..

[90] Bronsky J., Campoy C., Braegger C., Cai W., Carnielli V., Darmaun D., Decsi T., Domellöf M., Embleton N., Fewtrell M. (2018). ESPGHAN/ESPEN/ESPR/CSPEN guidelines on pediatric parenteral nutrition: Vitamins. Clin. Nutr..

[91] Domellöf M., Szitanyi P., Simchowitz V., Franz A., Mimouni F., Braegger C., Bronsky J., Cai W., Campoy C., Carnielli V. (2018). ESPGHAN/ESPEN/ESPR/CSPEN guidelines on pediatric parenteral nutrition: Iron and trace minerals. Clin. Nutr..

[92] Agostoni C., Buonocore G., Carnielli V., De Curtis M., Darmaun D., Decsi T., Domellöf M., Embleton N., Fusch C., Genzel-Boroviczeny O. (2010). Enteral Nutrient Supply for Preterm Infants: Commentary FROM the European Society of Paediatric Gastroenterology, Hepatology and Nutrition Committee on Nutrition. J. Pediatr. Gastroenterol. Nutr..

[93] Denne S.C. (2001). Energy Expenditure in Infants with Pulmonary Insufficiency: Is There Evidence for Increased Energy Needs?. J. Nutr..

[94] El Koofy N.M., Rady H.I., Abdallah S.M., Bazaraa H.M., Rabie W.A., El-Ayadi A.A. (2019). The effect of high fat dietary modification and nutritional status on the outcome of critically ill ventilated children: Single-center study. Korean J. Pediatr..

[95] White A.M., Liu P., Yee K., Waber B., Monk H.M., Zhang H., Jensen E.A. (2015). Determinants of Severe Metabolic Bone Disease in Very Low-Birth-Weight Infants with Severe Bronchopulmonary Dysplasia Admitted to a Tertiary Referral Center. Am. J. Perinatol..

[96] Hicks P.D., Rogers S.P., Hawthorne K.M., Chen Z., Abrams S.A. (2011). Calcium Absorption in Very Low Birth Weight Infants with and without Bronchopulmonary Dysplasia. J. Pediatr..

[97] Abrams S.A., Bhatia J.J.S., Corkins M.R., De Ferranti S.D., Golden N.H., Silverstein J., The Committee on Nutrition (2013). Calcium and Vitamin D Requirements of Enterally Fed Preterm Infants. Am. Acad. Pediatr..

[98] Mimouni F.B., Mandel D., Lubetzky R., Senterre T. (2014). Calcium, Phosphorus, Magnesium and Vitamin D Requirements of the Preterm Infant. World Rev. Nutr. Diet..

[99] Duan J., Kong X., Li Q., Hua S., Zhang S., Zhang X., Feng Z. (2016). Association between anemia and bronchopulmonary dysplasia in preterm infants. Sci. Rep..

[100] Rao R., Georgieff M.K. (2009). Iron Therapy for Preterm Infants. Clin. Perinatol..

[101] Dutta S., Singh B., Chessell L., Wilson J., Janes M., McDonald K., Shahid S., Gardner V.A., Hjartarson A., Purcha M. (2015). Guidelines for Feeding Very Low Birth Weight Infants. Nutrients.

[102] Park J.S., Jeong S.-A., Cho J.Y., Seo J.-H., Lim J.Y., Woo H.O., Youn H.-S., Park C.-H. (2020). Risk Factors and Effects of Severe Late-Onset Hyponatremia on Long-Term Growth of Prematurely Born Infants. Pediatr. Gastroenterol. Hepatol. Nutr..

[103] Huang J., Zhang L., Tang J., Shi J., Qu Y., Xiong T., Mu D. (2018). Human milk as a protective factor for bronchopulmonary dysplasia: A systematic review and meta-analysis. Arch. Dis. Child. Fetal Neonatal Ed..

[104] Friel J.K., Martin S.M., Langdon M., Herzberg G.R., Buettner G.R. (2002). Milk from Mothers of Both Premature and Full-Term Infants Provides Better Antioxidant Protection than Does Infant Formula. Pediatr. Res..

[105] Collado M.C., Santaella M., Mira-Pascual L., Martínez-Arias E., Khodayar-Pardo P., Ros G., Martínez-Costa C. (2015). Longitudinal Study of Cytokine Expression, Lipid Profile and Neuronal Growth Factors in Human Breast Milk from Term and Preterm Deliveries. Nutrients.

[106] Villamor-Martínez E., Pierro M., Cavallaro G., Mosca F., Villamor E. (2019). Mother’s Own Milk and Bronchopulmonary Dysplasia: A Systematic Review and Meta-Analysis. Front. Pediatr..

[107] Villamor-Martínez E., Pierro M., Cavallaro G., Mosca F., Kramer B.W., Villamor E. (2018). Donor Human Milk Protects against Bronchopulmonary Dysplasia: A Systematic Review and Meta-Analysis. Nutrients.

[108] Miller J., Tonkin E., Damarell R.A., McPhee A.J., Suganuma M., Suganuma H., Middleton P.F., Makrides M., Collins C.T. (2018). A Systematic Review and Meta-Analysis of Human Milk Feeding and Morbidity in Very Low Birth Weight Infants. Nutrients.

[109] Arslanoglu S., Boquien C.-Y., King C., Lamireau D., Tonetto P., Barnett D., Bertino E., Gaya A., Gebauer C., Grovslien A. (2019). Fortification of Human Milk for Preterm Infants: Update and Recommendations of the European Milk Bank Association (EMBA) Working Group on Human Milk Fortification. Front. Pediatr..

[110] Radmacher P.G., Adamkin D.H. (2017). Fortification of human milk for preterm infants. Semin. Fetal Neonatal Med..

[111] Macedo I., Pereira-Da-Silva L., Cardoso M. (2018). The fortification method relying on assumed human milk composition overestimates the actual energy and macronutrient intakes in very preterm infants. Matern. Health Neonatol. Perinatol..

[112] Pereira-Da-Silva L., Cardoso M., Macedo I. (2018). Associations of Measured Protein and Energy Intakes with Growth and Adiposity in Human Milk-Fed Preterm Infants at Term Postmenstrual Age: A Cohort Study. Am. J. Perinatol..

[113] Arslanoglu S., Moro G.E., Ziegler E.E. (2006). Adjustable fortification of human milk fed to preterm infants: Does it make a difference?. J. Perinatol..

[114] Morlacchi L., Mallardi D., Giannì M.L., Roggero P., Amato O., Piemontese P., Consonni D., Mosca F. (2016). Is targeted fortification of human breast milk an optimal nutrition strategy for preterm infants? An interventional study. J. Transl. Med..

[115] Rochow N., Fusch G., Ali A., Bhatia A., So H.Y., Iskander R., Chessell L., el Helou S., Fusch C. (2021). Individualized target fortification of breast milk with protein, carbohydrates, and fat for preterm infants: A double-blind randomized controlled trial. Clin. Nutr..

[116] Cardoso M., Virella D., Macedo I., Silva D., Pereira-Da-Silva L. (2021). Customized Human Milk Fortification Based on Measured Human Milk Composition to Improve the Quality of Growth in Very Preterm Infants: A Mixed-Cohort Study Protocol. Int. J. Environ. Res. Public Health.

[117] McLeod G., Sherriff J., Hartmann P.E., Nathan E., Geddes D., Simmer K. (2015). Comparing different methods of human breast milk fortification using measured v. assumed macronutrient composition to target reference growth: A randomised controlled trial. Br. J. Nutr..

[118] Shaikhkhalil A.K., Curtiss J., Puthoff T.D., Valentine C.J. (2014). Enteral Zinc Supplementation and Growth in Extremely-Low-Birth-Weight Infants with Chronic Lung Disease. J. Pediatr. Gastroenterol. Nutr..

[119] Guimarães H., Rocha G., Guedes M.B., Guerra P., Silva A.I., Pissarra S. (2014). Nutrition of preterm infants with bronchopulmonary dysplasia after hospital discharge—Part I. J. Pediatr. Neonatal Individ. Med..

[120] Bott L., Béghin L., Devos P., Pierrat V., Matran R., Gottrand F. (2006). Nutritional Status at 2 Years in Former Infants with Bronchopulmonary Dysplasia Influences Nutrition and Pulmonary Outcomes During Childhood. Pediatr. Res..

[121] Hay W.W., Hendrickson K.C. (2017). Preterm formula use in the preterm very low birth weight infant. Semin. Fetal Neonatal Med..

[122] Brunton J.A., Saigal S., Atkinson S.A. (1998). Growth and body composition in infants with bronchopulmonary dysplasia up to 3 months corrected age: A randomized trial of a high-energy nutrient-enriched formula fed after hospital discharge. J. Pediatr..

[123] Romera G., Figueras J., Rodríguez-Miguélez J.M., Ortega J., Jiménez R. (2004). Energy Intake, Metabolic Balance and Growth in Preterm Infants Fed Formulas with Different Nonprotein Energy Supplements. J. Pediatr. Gastroenterol. Nutr..

[124] Pereira-Da-Silva L., Dias M.P.G., Virella D., Moreira A.C., Serelha M. (2007). Osmolality of preterm formulas supplemented with nonprotein energy supplements. Eur. J. Clin. Nutr..

[125] Moltu S.J., Bronsky J., Embleton N., Gerasimidis K., Indrio F., Köglmeier J., de Koning B., Lapillonne A., Norsa L., Verduci E. (2021). ESPGHAN Committee on Nutrition. Nutritional management of the critically ill neonate: A Position Paper of the ESPGHAN Committee on Nutrition. J. Pediatr. Gastroenterol. Nutr..

[126] Konnikova Y., Zaman M.M., Makda M., D’Onofrio D., Freedman S.D., Martin C.R. (2015). Late Enteral Feedings Are Associated with Intestinal Inflammation and Adverse Neonatal Outcomes. PLoS ONE.

[127] Ambalavanan N., Carlo W.A., D’Angio C.T., McDonald S., Das A., Schendel D., Thorsen P., Higgins R.D. (2009). For the Eunice Kennedy Shriver National Institute of Child Health and Human Development Neonatal Research Network Cytokines Associated with Bronchopulmonary Dysplasia or Death in Extremely Low Birth Weight Infants. Am. Acad. Pediatr..

[128] Jadcherla S.R., Gupta A., Fernandez S., Nelin L.D., Castile R., Gest A.L., Welty S. (2008). Spatiotemporal Characteristics of Acid Refluxate and Relationship to Symptoms in Premature and Term Infants with Chronic Lung Disease. Am. J. Gastroenterol..

[129] Wang L.-J., Hu Y., Wang W., Zhang C.-Y., Bai Y.-Z., Zhang S.-C. (2020). Gastroesophageal reflux poses a potential risk for late complications of bronchopulmonary dysplasia: A prospective cohort study. Chest.

[130] Nobile S., Noviello C., Cobellis G., Carnielli V.P. (2015). Are Infants with Bronchopulmonary Dysplasia Prone to Gastroesophageal Reflux? A Prospective Observational Study with Esophageal pH-Impedance Monitoring. J. Pediatr..

[131] Malcolm W.F., Smith P.B., Mears S., Goldberg R.N., Cotten C.M. (2009). Transpyloric tube feeding in very low birthweight infants with suspected gastroesophageal reflux: Impact on apnea and bradycardia. J. Perinatol..

[132] A Jensen E., Zhang H., Feng R., Dysart K., Nilan K., A Munson D., Kirpalani H. (2019). Individualising care in severe bronchopulmonary dysplasia: A series of N-of-1 trials comparing transpyloric and gastric feeding. Arch. Dis. Child. Fetal Neonatal Ed..

[133] Reynolds E.W., Grider D., Caldwell R., Capilouto G., Patwardhan A., Charnigo R. (2018). Effects of Bronchopulmonary Dysplasia on Swallow: Breath Interaction and Phase of Respiration with Swallow During Non-nutritive Suck. S. Pac. J. Nat. Appl. Sci..

[134] Luo J., Shepard S., Rn K.N., Wood A., Monk H.M., Jensen E.A., Harrington A.T., Maschhoff K., Kirpalani H., Feng Z. (2018). Improved growth and developmental activity post tracheostomy in preterm infants with severe BPD. Pediatr. Pulmonol..

[135] Guimarães H., Rocha G., Guedes M.B., Guerra P., Silva A.I., Pissarra S. (2014). Nutrition of preterm infants with bronchopulmonary dysplasia after hospital discharge—Part II. J. Pediatr. Neonatal Individ. Med..

[136] A Morgan J., Young L., McCormick F.M., McGuire W. (2011). Promoting growth for preterm infants following hospital discharge. Arch. Dis. Child. Fetal Neonatal Ed..

[137] Marino L.V., Fudge C., Pearson F., Johnson M.J. (2019). Home use of breast milk fortifier to promote postdischarge growth and breast feeding in preterm infants: A quality improvement project. Arch. Dis. Child..

[138] Young T.E. (2007). Nutritional support and bronchopulmonary dysplasia. J. Perinatol..

[139] Villa E., Barachetti R., Barbarini M. (2017). Nutritional management of preterm newborn after hospital discharge: Energy and nutrients. La Pediatr. Medica Chir..

[140] Pereira-Da-Silva L., Virella D., Fusch C. (2019). Nutritional Assessment in Preterm Infants: A Practical Approach in the NICU. Nutrients.

[141] Landau-Crangle E., Rochow N., Fenton T.R., Liu K., Ali A., So H.Y., Fusch G., Marrin M.L., Fusch C. (2018). Individualized Postnatal Growth Trajectories for Preterm Infants. J. Parenter. Enter. Nutr..

[142] Fenton T.R., Kim J.H. (2013). A systematic review and meta-analysis to revise the Fenton growth chart for preterm infants. BMC Pediatr..

[143] Pereira-Da-Silva L., Virella D. (2014). Is intrauterine growth appropriate to monitor postnatal growth of preterm neonates?. BMC Pediatr..

[144] Rochow N., Raja P., Liu K., Fenton T., Landau-Crangle E., Göttler S., Jahn A., Lee S., Seigel S., Campbell D. (2016). Physiological adjustment to postnatal growth trajectories in healthy preterm infants. Pediatr. Res..

[145] Pereira-da-Silva L., Virella D., Frutuoso S., Cunha M., Rocha G., Pissarra S. (2020). Recommendation of charts and reference values for assessing growth of preterm infants: Update by the Portuguese Neonatal Society. Port. J. Pediatr..

[146] Fenton T., Senterre T., Griffin I.J. (2018). Time interval for preterm infant weight gain velocity calculation precision. Arch. Dis. Child. Fetal Neonatal Ed..

[147] Brennan A.-M., Murphy B.P., Kiely M.E. (2016). Optimising preterm nutrition: Present and future. Proc. Nutr. Soc..

[148] Johnson M.J., Wiskin A.E., Pearson F., Beattie R.M., Leaf A.A. (2014). How to use: Nutritional assessment in neonates. Arch. Dis. Child. Educ. Pract. Ed..

[149] Visser F., Sprij A.J., Brus F. (2012). The validity of biochemical markers in metabolic bone disease in preterm infants: A systematic review. Acta Paediatr..

[150] Villar J., Giuliani F., Bhutta Z.A., Bertino E., Ohuma E.O., Ismail L.C., Barros F.C., Altman D.G., Victora C., Noble J.A. (2015). Postnatal growth standards for preterm infants: The Preterm Postnatal Follow-up Study of the INTERGROWTH-21 st Project. Lancet Glob. Health.

